# Epstein–Barr Virus-Induced Metabolic Rearrangements in Human B-Cell Lymphomas

**DOI:** 10.3389/fmicb.2018.01233

**Published:** 2018-06-08

**Authors:** Pier P. Piccaluga, Alessandra Weber, Maria R. Ambrosio, Yonis Ahmed, Lorenzo Leoncini

**Affiliations:** ^1^Department of Experimental, Diagnostic, and Specialty Medicine, Bologna University School of Medicine, Bologna, Italy; ^2^Euro-Mediterranean Institute of Science and Technology, Palermo, Italy; ^3^Department of Pathology, Jomo Kenyatta University of Agriculture and Technology, Juja, Kenya; ^4^Section of Pathology, Department of Medical Biotechnology, University of Siena, Siena, Italy; ^5^Medical Research Institute, Alexandria University, Alexandria, Egypt

**Keywords:** Epstein-Barr virus, EBV, lymphoma, metabolism, review, MYC

## Abstract

Tumor metabolism has been the object of several studies in the past, leading to the pivotal observation of a consistent shift toward aerobic glycolysis (so-called Warburg effect). More recently, several additional investigations proved that tumor metabolism is profoundly affected during tumorigenesis, including glucose, lipid and amino-acid metabolism. It is noticeable that metabolic reprogramming can represent a suitable therapeutic target in many cancer types. Epstein–Barr virus (EBV) was the first virus linked with cancer in humans when Burkitt lymphoma (BL) was described. Besides other well-known effects, it was recently demonstrated that EBV can induce significant modification in cell metabolism, which may lead or contribute to neoplastic transformation of human cells. Similarly, virus-induced tumorigenesis is characterized by relevant metabolic abnormalities directly induced by the oncoviruses. In this article, the authors critically review the most recent literature concerning EBV-induced metabolism alterations in lymphomas.

## Introduction

Oncogenic viruses are an important public health issue since they are responsible for 20% of total human cancer cases. The seven following viruses are currently considered proper human oncogenic viruses: hepatitis B and C virus (HBV and HCV) account for approximately 80% of cases of hepatocellular carcinoma (HCC) worldwide. Human papillomavirus (HPV, 16 and 18 especially) is implied in cervical cancer, anogenital neoplasms, and head and neck tumors. Human herpes virus 8 (HHV8) is responsible for AIDS-related Kaposi’s sarcoma. Merkel cell polyomavirus (MCPyV), causes Merkel cell carcinoma. HTLV-1 is the agent causing adult T-cell lymphoma. Epstein–Barr virus (EBV), finally, is associated with many tumors including nasopharyngeal carcinoma (NPC) and different lymphoma subtypes (**Table 1**; Luo and Ou, 2015).

**Table 1:** Association between oncoviruses and lymphomas.

Disease	Oncovirus	Experimentally proven metabolic abnormality	Main Postulated involved oncogene	Reference
Post-transplant lymphoproliferative disease	EBV	No	MYC	Cesarman, 2011; Nourse et al., 2011
Diffuse large B-cell lymphoma^∗^	EBV	No	NF-kB	Kanno et al., 1997; Dojcinov et al., 2011; Kato et al., 2014
Burkitt lymphoma	EBV	Yes: increased import of key nutrients, increased fatty acid synthesis and glycolysis	MYC	Yoon et al., 2007; Daye and Wellen, 2012; Dang, 2013; Eberlin et al., 2014; Altman et al., 2016
Hodgkin lymphoma	EBV	No	NF-kB	Chapman and Rickinson, 1998; Kuppers, 2009
Primary effusion lymphoma	HHV-8+/EBV±	No	PI3K/AKT	Okada et al., 2014; Mediani et al., 2016
Multicentric Castleman disease	HHV-8+	No	Not known	Wang et al., 2016
Adult T-cell leukemia/lymphoma	HTLV-1	No	NF-kB	Bangham and Ratner, 2015
EBV-positive T-cell lymphoproliferative disorders	EBV	No	PI3K/AKT	Anagnostopoulos et al., 1992; Ho et al., 1999; Au et al., 2004; Zhou et al., 2007; Cai et al., 2015

*Adapted from “Immunodeficiency-associated viral oncogenesis” by A. Pierangeli, G. Antonelli and G. Gentile, 2015, Clinical Microbiology and Infection, Volume 21, Issue 11, 975 – 983. EBV, Epstein–Barr virus; HHV-8, human herpesvirus-8; HTLV-1, human T-cell lymphotropic virus type 1. ^∗^Not in the post-transplant setting. Based on the expression of virus-encoded molecules proved to be an effect in experimental model*.

These oncogenic viruses exert their oncogenic power by interfering with multiple molecular signaling pathways. Sometimes they directly integrate their DNA in the host cell one [as does HPV in cervical cancer cells (Fang et al., 2014) or HBV in hepatocytes inducing HCC development (Herrmann et al., 1995)], some others they alter the expression of miRNAs (Ambrosio et al., 2014b), and in other cases, they directly interact with proteins expressed by normal or cancer cells (this is the most important pathogenetic way by which HCV induces HCC; Tsai and Chung, 2010). Of these changes, certain ones may induce chronic inflammation, and others can disrupt the cellular genetic and epigenetic integrity (Fang et al., 2014) or interfere with the host cell homeostasis and DNA repair mechanisms, thus determining genome instability and deregulating the cell cycle (Luo and Ou, 2015). Some of these changes are strictly connected with metabolic dysfunction, such as enhanced glucose uptake and glycolysis, dysregulation of molecular pathways regulating oxidative stress, or alterations of lipid metabolic expression patterns (Luo and Ou, 2015; Lo et al., 2017).

The link between metabolic alterations and cancerogenesis, even when not caused by oncogenic viruses, is still under investigation. It is certain that the tumor cell goes through a metabolic rearrangement, such as an augmented glucose capture, and many metabolic patterns have proven to be altered in cancer cells in linkage to oncogenes’ mutations (Lo et al., 2017). A number of oncogenes, such as *MYC*, hypoxia inducible factors-1 alpha subunit (*HIF1A*), and tumor suppressor p53 (Yeung et al., 2008) as well as some pathways, such as phosphoinositide 3-kinase (PI3K/AKT) and protein kinase B (PKB), or the mammalian target of rapamycin (*MTORC1*) one (Wieman et al., 2007), are known to be involved in the energy metabolism regulation of cancer cells.

The interaction between tumor and metabolism of the cell may be direct, via the increase of nutrients that are available for tumor growth (for example, by interacting with glucose transporters and glycolytic enzymes). It may also be indirect, via the modulation of cell proliferation pathways, such as the PI3K and mitogen-activated protein kinase (MAPK), or stress response factors, such as HIF1A and 5′ AMP-activated protein kinase (AMPK), that are related to metabolic control (Chen and Russo, 2012; Noch and Khalili, 2012).

The study of these mechanisms may be useful not only to fully understand the pathogenesis of virus-induced tumors, but also to widen the range of therapeutic options, combining anti-viral and antiglycolytic or anti-lipid therapies in the cure of virus-driven cancers.

In this review, we are going to focus on how EBV infected cells are more likely to develop cancer. We will consider in particular some types of cancers, and how metabolic dysregulation can be one of the possible ways EBV employs to drive cancerogenesis in these cells.

## Epstein–Barr Virus and Cancerogenesis

The EBV is a lymphocryptovirus of the γ-herpesvirus family. It affects up to 90% of human adult population, typically establishing a life-long persistent infection. It is considered a group 1 carcinogen, according to the International Agency for Research on Cancer, for its remarkable association with a large variety of lymphoid and epithelial malignancies. According to unadjusted estimates, nearly 3.7 million people are suffering from EBV-driven cancers (de Martel et al., 2012). EBV has been detected not only in Burkitt’s lymphoma (Navari et al., 2015), in which the virus itself was discovered, and NPC, in which it is almost always present, but it is also being related to Hodgkin lymphoma, post-transplant lymphoproliferative disorders (PTLD), T-cell non-Hodgkin lymphomas (NHL), and gastric carcinomas. The evidence of association with breast, HCCs, and smooth muscle cell-derived tumors in immunodeficient individuals is less clear (Niedobitek et al., 2001). Probably – since a pathogenetic role of EBV has been demonstrated in so many cancers and the virus itself is so widespread – it is not impossible to think that a certain percentage of cases of any type of cancer might conceal the presence of EBV infection; nevertheless, B lymphocytes and epithelial cells remain its preferential host cells.

The association with EBV can depend for most of these tumors on geographical factors [e.g., Burkitt lymphoma (BL)], histological subtype of the tumor (e.g., Hodgkin lymphoma), or the cellular subtype infected (e.g., in T-cell lymphomas usually only some of the cells are infected; Niedobitek et al., 2001).

The pathogenetic role of EBV in cancer development is complex and yet not fully understood. An important role is for sure played by latency products; these are expressed for the most part of the virus lifetime, that is to say when the virus is not in its lytic cycle replication, but in its latent phase. These affect genes which are necessary for the immortalization of B-cells, by permanently inducing the physiological molecular pathways that lead to B-cell activation and cell division. These are the Epstein–Barr nuclear antigen (EBNA) 1, 2, -LP, 3A, 3B, and 3C and the latent membrane protein (LMP) 1, 2A, and 2B protein families. Furthermore, two small (170 bases in length) non-polyadenylated non-coding nuclear RNAs, the EBV encoded small RNAs 1 and 2 (EBER and EBER2), are abundantly expressed in latent infection, up to 10^7^ copies per cell (Niedobitek et al., 2001).

Based on how these genes are expressed in different combinations in EBV-infected cells, three possible latency patterns have been identified (**Table 2**).

**Table 2:** Canonical EBV latency patterns.

	Latency
	
	I	II	III
EBERs	+	+	+
EBNA1	+	+	+
EBNA2	-	-	+
EBNA3, A, B, C	-	-	+
EBNA-LP	-	-	+
LMP1	-	+	+
LMP2, A,B	-	+	+
miRNAs	BART miRNAs (modest)	BART miRNAs (high)	BHRF1 miRNAs (high) BART miRNAs (modest)

Remarkably, recent evidence suggests that atypical latency patterns are common, at least in lymphoid tumors (Abate et al., 2015). Probably, the most commonly implicated in cancerogenesis gene products are EBNA2 and LMP1 (Young and Rickinson, 2004).

## Metabolic Alterations in Tumors

Cells can depend for their energy supply on two different major pathways: oxidative phosphorylation and extra-mitochondrial glycolysis. Usually, healthy and normally vascularized cells tend to rely mostly on oxidative phosphorylation for ATP synthesis, since this pathway is more effective in terms of energy supply, while they tend to depend on glycolysis only in conditions of hypoxia. In cancers, however, cells typically tend to increase the extra-mitochondrial glycolysis. Even in the presence of oxygen, they usually rely on this metabolic pathway for ATP synthesis, rather than on oxidative phosphorylation. This metabolic reprogramming process is known as the Warburg effect (Warburg et al., 1927; Gatenby and Gillies, 2004). Not only it allows malignant cells’ survival even in conditions of hypoxia, but also it contributes to cell proliferation: the energy that is not converted into ATP can in fact be shifted toward the constitution of various macromolecules (proteins, nucleic acids, and lipids), which are useful to the cell growth (Jones and Bianchi, 2015). Moreover, the decrease of oxidative phosphorylation also probably leads to a less efficient reactive oxygen species (ROS) production, such as hydrogen peroxide and superoxide anion, which can be harmful to the cell or promote cellular senescence (Kim and Dang, 2006).

Not only do cancer cells tend to use glucose differently from normal cells, but they also need more of it for their rapid growth: the number of glucose transporters on the cell membrane is typically increased. The avid uptake of glucose by tumors is the rationale for their detection by fluorodeoxyglucose positron emission tomography (FDG-PET; Macheda et al., 2005).

Finally, lipid metabolism too has proven to be altered in many cancer types: first, fatty acids, phospholipids, and cholesterol, the three main lipid molecules of our cells, are significantly increased and actively biosynthesized in tumor cells. Second, choline kinase, an enzyme playing a key role in the biosynthesis of phosphatidylcholine, is upregulated in multiple cancer cell lines (Janardhan et al., 2006) and, third, active sterol biosynthesis helps cell proliferation (Huang and Freter, 2015). Moreover, there is a significant overexpression of genes involved in cholesterol biosynthetic pathway in refractory tumors (Krycer and Brown, 2013; Huang and Freter, 2015).

Many findings suggest a correlation between EBV infection and some of these metabolic alterations. During EBV infection, the virus meets the amplified bioenergetic and biosynthetic demands, by hijacking host cell metabolism to increase nutrient uptake and anabolic metabolism. Doing this, it mirrors the Warburg effect normally occurring in highly proliferating and neoplastic cells. Nevertheless, unlike cancer cells, EBV undergoes powerful selection for efficiency, and has the capacity to rapidly and strongly reprogram host cell metabolism (Cuninghame et al., 2014; Mesri et al., 2014; Pierangeli et al., 2015; Levy and Bartosch, 2016). Particularly, EBV can affect glucose and lipid metabolism, while altering enzymatic functions (e.g., HIF1A and AMPK) and inducing ROSproduction (Lo et al., 2017).

## EBV-Driven Carbohydrate Metabolic Alterations in Nasopharyngeal Carcinoma (NPC)

**FIGURE 1:**
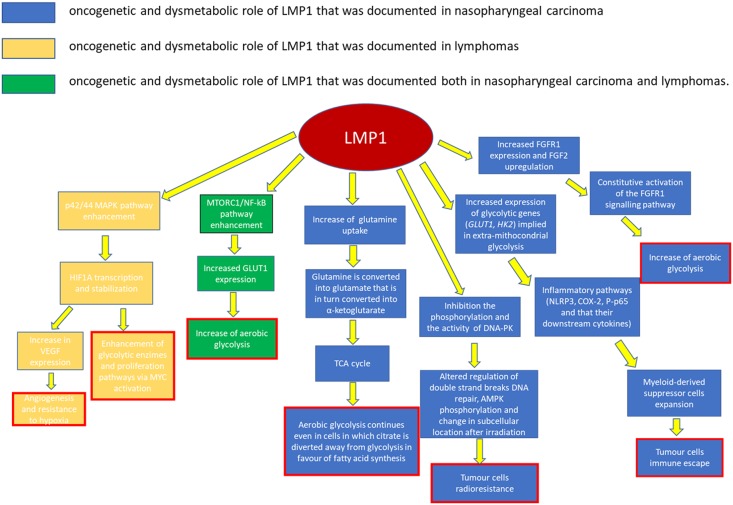
Role played by LMP1 in cancerogenesis.

Ninety-five percent of NPC cases in South China (where this cancer is most commonly diagnosed) are of the histological type III according to the WHO classification (undifferentiated type), which is associated with EBV infection (Raab-Traub, 2002). In the EBV-infected NPC cells, the most common latency program is latency II type. This includes the expression of LMP1 and LMP2, EBNA1, EBERs, as well as some EBV-encoded miRNAs (**Table 2**). Among these antigens, LMP1 is considered as a crucial oncoprotein (**Figure 1**), as it is implicated in the maintenance of latent infection and in the malignant transformation. LMP1 has also a role in the metastatic and local invasion, enhancing the production of angiogenic factors, as well as in the processes linked to inflammation and antigen presentation, which may favor the tumor progression, but also its immune escape ability (Hitt et al., 1989; Dawson et al., 2012). Myeloid-derived suppressor cells (MDSCs) are key regulatory cells that have a role in this latter process, as they are physiologically appointed to inflammation control but are also able to favor immune escape (Cai et al., 2017).

The microenvironment of a wide range of tumors, including NPC, has shown to be enriched in MDSCs. EBV may be implied in their expansion, though it is not clear how yet. One possible hypothesis is that LMP1 promotes MDSC expansion by increasing the rate of extra-mitochondrial glycolysis in cancer cells. This is consistent with the observation that a high expression level of LMP1, glucose transporter 1 (GLUT1), and CD33+MDSCs is frequently reported in NPC sections (Cai et al., 2017).

Recent studies have confirmed that LMP1 prompts the so-called “aerobic glycolysis” by inducing the expression of multiple glycolytic genes, including *GLUT1* and hexokinase 2 (*HK2*; Lo et al., 2015). One other way LMP1 increases GLUT1 levels is by blocking its K48-ubiquitination and p62-dependent autolysosomal degradation, thus stabilizing the protein (Cai et al., 2017). The increase in glucose consumption and in the production of lactate suggests that the augmented glucose avidity is almost entirely used to implement extra-mitochondrial glycolysis.

This metabolic reprogramming induces an expansion of the MDSC subset mainly by favoring some inflammatory pathways. The expression of the Nod-like receptor family protein 3 (NLRP3) inflammasome is increased, as well as COX-2 and P-p65 ones, so that their downstream cytokines (IL-1β, IL-6, and GM-CSF) are also increased (Cai et al., 2017).

Other current findings have implied that LMP1 is also involved in the fibroblast growth factor 2 and fibroblast growth factor receptor 1 (FGF2/FGFR1) pathway activity, which can also contribute to the increase of aerobic glycolysis in the EBV-infected epithelial, and in turn lead them to evolve in NPC (Lo et al., 2015).

Constitutive FGFR1 activation, due to mutation or overexpression of FGFR1 or its ligands, is known to induce cellular transformation and has been documented in a variety of human malignancies (Kelleher et al., 2013). This activation is common in NPC, especially in cases that are LMP1-positive, i.e., the EBV-infected ones. With respect to these cases, LMP1 leads to the constitutive activation of the FGFR1 signaling pathway, not only by increasing FGFR1 expression, but also by upregulating FGF2 (Lo et al., 2017). The role of this pathway in cancer transformation and growth is confirmed by the fact that FGFR1 inhibitors attenuate both LMP1-mediated aerobic glycolysis, reducing lactate dehydrogenase A (LDHA) phosphorylation and activity in nasopharyngeal epithelial cells, and cellular transformation, proliferation, migration, and invasion (Kelleher et al., 2013; Lo et al., 2015).

GLUT1 upregulation via MTORC1/*nuclear factor kappa-light-chain-enhancer of activated B cells* (NF-kB) signaling pathways is another main oncogenic pathway of EBV in NPC cells (Wieman et al., 2007; Lo et al., 2017). MTORC1 is a serine/threonine protein kinase pathway that is evolutionarily conserved. It is involved in regulating energy metabolism and cell growth by controlling protein translation and ribosome biogenesis (Zhang et al., 2017). It is usually activated by PI3K/AKT and other phosphorylating pathways. In EBV-infected cells, LMP1 activates MTORC1 which, in turn, upregulates the expression of *GLUT1*. C-terminal activating region 2 (CTAR2) of LMP1 is probably the main domain involved in MTORC1 activation, through an IKK-mediated process (phosphorylation of TSC2 at Ser939; Zhang et al., 2017).

It has been demonstrated that disrupting the activity of MTORC1 signaling in EBV-driven NPC effectively suppresses both LMP1-induced NF-kB activation and *GLUT1* transcription. On the other hand, blocking NF-kB signaling preserves MTORC1 activity but alters GLUT1 transcription. This result suggests that GLUT1 is a direct target of NF-kB signaling (Lo et al., 2017; Zhang et al., 2017).

LMP1 seems to be implied also in the process of DNA damage response (DDR), especially when involved in radio-resistance. HK2 expression levels are positively correlated to LMP1 expression in NPC tissues, and they are associated with poor survival rates after tumor radiation (Xiao et al., 2014; Lo et al., 2017). In particular, LMP1 can inhibit the phosphorylation and the activity of the DNA-dependent protein kinase (DNA-PK), a key enzyme for the double-strand breaks (DSBs) repair (Lu et al., 2016; Lo et al., 2017). Together with other oncoproteins (EBNA1 and EBNA2), LMP1 can also increase the cellular levels of ROS and alter the mitotic checkpoint (Gruhne et al., 2009). As a matter of fact, ROS levels fluctuate (due to inefficient oxidative phosphorylation) in EBV-infected cells. Increases or decreases in the level of ROS seem to favor carcinogenesis (Gruhne et al., 2009; Lu et al., 2016).

Moreover, by disrupting the physical interaction between AMPK and DNA-PK, LMP1 can reduce AMPK phosphorylation and change its subcellular location after irradiation (Lu et al., 2016). AMPK is a crucial energy sensor that, as a negative regulator of glycolysis, helps the maintenance of cellular energy homeostasis. However, it is also a regulator of the DDR pathway in response to genomic stress. A decrease in AMPK activity is associated with resistance to irradiation-induced apoptosis, and thus to a poorer clinical outcome in NPC patients treated with radiation therapy. At the same time, the reactivation of AMPK, which may be obtained via AMPK activators, significantly promotes radiosensitivity and could be efficiently used for facilitating NPC radiotherapy (Lu et al., 2016).

## EBV-Driven Carbohydrate Metabolic Alterations in Lymphomas

EBV is a powerful lymphocyte growth-promoter and transformer. It is etiologically linked to many lympho-proliferative disorders and malignant lymphomas including BL, Hodgkin lymphoma, some types of diffuse large B cell lymphomas, and rare B lymphomas of the immunocompromised state, as well as T/NK cell lymphoproliferative disorders, for example, extranodal NK/T-cell lymphoma, nasal type (Shannon-Lowe et al., 2017).

**FIGURE 2:**
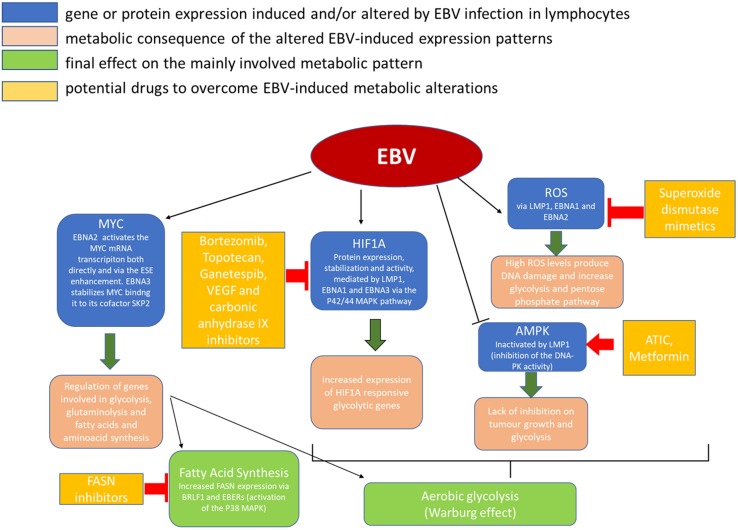
Metabolic alterations induced in cancer cells by EBV infection and possible therapeutic targets.

The part played by EBV in these tumors’ pathogenesis shares some common points with that of EBV in NPC, including the mostly LMP1-driven metabolic disruption, which plays an important role in this case as well (**Figure 2**).

Glucose consumption, a hallmark of the Warburg effect, is common to antigen or mitogen stimulated lymphocytes and many B-lymphomas. This suggests the importance of a glycolysis-based mechanism to support rapid lymphocyte proliferation (Sariban-Sohraby et al., 1983; Sommermann et al., 2011). The way this mechanism works is now beginning to be understood (Sariban-Sohraby et al., 1983) and it is becoming clear how some key genes (such as PI3K and TP53) probably play a crucial role in it (Calvo-Vidal and Cerchietti, 2013).

Moreover, recent findings suggest that, especially during cell proliferation and stress conditions, other substrates, besides glucose, can become an important driving force for glycolysis, this including nucleotide biosynthesis and glycine-folate metabolism (Calvo-Vidal and Cerchietti, 2013).

As for nucleotide biosynthesis, one of the major requirements for cell proliferation is the production of DNA and RNA which needs the incorporation of about ten carbon atoms for each nucleotide (9 for pyrimidines and 10 for purines, respectively; Calvo-Vidal and Cerchietti, 2013). Five of these atoms are derived from the ribose-phosphate pathway. If an exogenous source of nucleotides is available, nucleotides can be obtained by the salvage pathway as well. Nevertheless, when there is an important increase in lymphocytes’ proliferation, the number of nucleotides required is so high that the *de novo* pathway must be activated (Calvo-Vidal and Cerchietti, 2013). One proof of this can be the increased activity of the inosine monophosphate dehydrogenase (IMPDH) activity, an enzyme catalyzing a fundamental reaction of *de novo* purine biosynthesis (nicotinamide adenine dinucleotide-dependent oxidation of inosine monophosphate to xanthosine monophosphate), which has been demonstrated in T-cell and B-cell leukemias and lymphomas (Hovi et al., 1976; Nagai et al., 1991; Calvo-Vidal and Cerchietti, 2013).

A possible source for purine synthesis is glycine, which can supply carbon both to the DNA itself (together with nitrogen) and to folates that are required for the synthesis of purines and thymidine. It has been shown that cancer cell proliferation is associated to *in vitro* glycine consumption (while its depletion prolongs the cell cycle) and that the upregulation of its endogenous mitochondrial production is required for cancer cells’ metabolic reprogramming (Jain et al., 2012; Calvo-Vidal and Cerchietti, 2013).

It has also been suggested that the glycine pathway plays a role in genetic and epigenetic cell integrity. Its alterations may then affect the response of lymphoma cells to therapy, both chemotherapy and epigenetic-targeted one (Calvo-Vidal and Cerchietti, 2013; Vazquez et al., 2013).

The NF-kB pathway has been hypothesized as another pathway involved in glucose import. NF-kB activation is typical of transformed B lymphocytes including Herpes virus transformed lymphoblasts, mimicking antigen co-receptor signaling in B-lymphocytes (Kawauchi et al., 2008; Krawczyk et al., 2010). NF-kB and its regulatory upstreaming protein *inhibitor of nuclear factor kappa B kinase subunit beta* (IKBKB) transcription can probably favor lymphoma cell survival by promoting the increase of GLUT proteins on the plasma membrane via AKT (Sommermann et al., 2011).

Glucose import across the lymphocyte cell membrane, in fact, is mostly facilitated by GLUT proteins, the levels of which can be regulated by some oncogenes and tumor suppressors. For example, MYC and RAS induce GLUT1 mRNA (Osthus et al., 2000), while p53 suppresses GLUT1, 3, and 4 expression (Schwartzenberg-Bar-Yoseph et al., 2004). PI3K instead increases both GLUT1 and GLUT3 expression (by inducing their mRNA through the HIF1A pathway), and GLUT4 translocation to the plasma membrane (moving from the storage vesicles under the effect of AKT activation) in B lymphocytes (Zelzer et al., 1998; Mîinea et al., 2005; Satoh, 2014) and probably in T-cells as well (Wieman et al., 2007).

Taken together, these findings suggest that NF-kB signaling, increasing the glucose import, supports proliferation and resistance of cancer cells (Sommermann et al., 2011) and that EBV has an important role in its activation (Gewurz et al., 2011). As a matter of fact, the inhibition of the NF-kB pathway in the EBV transformed B-cells lowers the glucose uptake to the point of triggering its autophagy-induced death. When NF-kB is inhibited, an alternate carbon source can overcome the effect on autophagy and cell death, whereas autophagy inhibitors accelerate them (Pujals et al., 2015).

HIF1A is another important factor which has been implied both in epithelial and B-cell derived cancers in EBV infection (Cuninghame et al., 2014). As the name says, this factor is typically expressed in hypoxic conditions by normal cells to adapt to the hostile environment. It can also be expressed in tumor cells enhancing glycolysis and activating MYC. In addition, the stabilization of HIF1A and its translocation to the nucleus can upregulate genes involved in cell growth, survival, and angiogenesis (Cuninghame et al., 2014).

It has been wondered whether the increased expression of HIF1A in cancer cells is a consequence of the necessity for adaptation to the hypoxic tumor microenvironment (Lo et al., 2013, 2015) or it is an intrinsic initiating event of the tumor development. The fact that EBV infected B cells show upregulation of HIF1A protein expression seems to validate the latter hypothesis (Wakisaka et al., 2004; Lo et al., 2013, 2015; Xiao et al., 2014; Sung et al., 2016). The proteins that increase HIF1A expression are different in the different latency patterns: LMP1 stimulates HIF1A transcriptional activity through the p42/44 MAPK pathway (Wakisaka et al., 2004; Lo et al., 2013, 2015; Sung et al., 2016) and contributes to its stabilization (Kondo et al., 2006; Lo et al., 2013, 2015); both LMP1 and EBNA-1 upregulate HIF1A and its downstream targets, IL-8 and VEGF (O’Neil et al., 2008; Lo et al., 2013, 2015, 2017); EBNA-3s, which are expressed in lymphatic chronic leukemia (LCL), bind the prolyl hydroxylase enzymes (PHD1 and PHD2), blocking their phosphorylating activity and thus stabilizing HIF1A (the complex HIF1, composed of HIF1A and ARNT, is regulated by proteolysis of its α-subunits, mediated by the oxygen-dependent hydroxylation of specific prolyl residues; Appelhoff et al., 2004; Darekar et al., 2012; Lo et al., 2013, 2015, 2017).

## Lipid Metabolism Alteration

Lipids are a wide group of diverse hydrophobic molecules including triacylglycerides, phosphoglycerides, sphingolipids, and sterols. Lipids are involved in cellular functions as both structural components and as signaling factors (Santos and Schulze, 2012). As for the structural role, fatty acids are used for the synthesis of triacylglycerides, while phosphoglycerides, sphingolipids, and sterols represent the principal components of the plasma membrane and of other cellular membranes (Santos and Schulze, 2012). As for the role in signaling, lipids can function as hormones and as second messengers (Santos and Schulze, 2012). Therefore, lipid metabolism abnormalities in cancer cells substantially affect membrane constitution, energy production, and cellular signaling (Santos and Schulze, 2012; Hashmi et al., 2015).

These alterations have been demonstrated both in NPC (Daker et al., 2013) and in lymphomas (Ambrosio et al., 2012).

One common lipid metabolism alteration in EBV infection is the increased expression of fatty acid synthase (FASN), an enzyme that plays a key role in the cell endogenous fatty acid synthesis (FAS) mediating the multiple condensation reaction between malonil-CoA and acetyl-CoA molecules which leads to the palmitate FAS (**Figure 2**). In particular, the transcription factor immediate-early (IE) protein BRLF1 (R) – an inductor of the lytic form of EBV infection – can activate FASN expression through a p38 stress MAPK-dependent mechanism. At the same time, BRLF1 can favor directly or indirectly the transcription of other early viral promoters, including the EBV IE gene, *BZLF1* (Z), that could have a role in increasing FAS too (Li et al., 2004).

*De novo* FAS is physiologically very active during the embryological and fetal life (especially in the lung development since FAs are used for synthesizing the surfactant) and in adult females under hormone stimulation during the menstrual cycle, being useful to the thickening of the endometrial wall, and to the supply of milk with fatty acids during lactation. As for the other tissues, a high-level FASN expression is normally limited to liver, brain, lung, and adipose tissue in adults, as the main lipid income for the cell comes from circulating lipids (Weiss et al., 1986; Pizer et al., 1997; Wagle et al., 1999; Kusakabe et al., 2000; Anderson et al., 2007). FASN expression can be increased, though, in human epithelial cells infected with EBV in its lytic form (Li et al., 2004) and in cancer cells (Lo et al., 2017). In cancer, fatty acids, whatever the levels of circulating triglycerides, are preferentially obtained by the activation of *FASN* gene products in order to engage a *de novo* synthesis of palmitate as opposed to what normally happens in healthy cells of well-nourished adult individuals, which tend to rely on fatty acids obtained from the diet (Menendez and Lupu, 2007; Lo et al., 2017). A FAS increase, mediated by FASN increased expression, has been demonstrated in breast cancer (especially HER2+ one; Yoon et al., 2007), prostate cancer (Swinnen et al., 2000), colorectal cancer (Li et al., 2000), and recently also in BL, where it seems to be related to EBERs’ expression levels (Ambrosio et al., 2012). One important confirmation to these findings is that the FASN-driven tumorigenesis in EBV-infected cells can be efficiently blocked by the use of FASN inhibitors (Li et al., 2004).

As we have seen, tumor cells shift their metabolism to aerobic glycolysis. However, aerobic glycolysis diverts citrate away from the mitochondrial Krebs, which is necessary for FAS. To promote aerobic glycolysis and FAS at the same time in rapidly proliferating cells, glutamine needs to be rapidly replenished into the tri-carboxylic acid (TCA) cycle (Lo et al., 2017). Glutamine is an amino acid which supplies alternative intermediates for TCA cycle. It is transported into cells through the SLC1A5 transporter, and then converted through an anaplerotic reaction to α-ketoglutarate (Daye and Wellen, 2012; Lo et al., 2013, 2015; Altman et al., 2016). It has been noticed that many cancer cells use glutamine for oxidative phosphorylation, FAS, and protein synthesis, and that γ-herpesviruses, including EBV, can induce glutaminolysis. Specifically, LMP1 was found to increase glutamine uptake and to induce elevate levels of intracellular glutamate in EBV-infected nasopharyngeal epithelial cells (Lo et al., 2013, 2015, 2017).

One key gene that has been related both to carbohydrate and to lipid metabolic dysregulation in lymphomas is *MYC* (Lo et al., 2017). *MYC* is a very well-known oncogene that contributes to tumorigenesis by regulating key genes implicated in patterns that converge to boost cellular growth and proliferation (Dang, 2013). Its effects can be both direct (increase of energetic supplies) and indirect (regulation of ribosomal and mitochondrial biosynthesis, glucose, glutamine, and lipid metabolism; Dang, 2013; Lo et al., 2017). Although the role of MYC in lymphomas’ lipids regulation has not been completely elucidated yet (Eberlin et al., 2014), the analysis of lipid pattern expression has shown different profiles between the MYC overexpressing ones and the others. How this is obtained is still under investigation: according to the most recent analyses, MYC could be implied in increasing the FAS and the cell supply of acetyl-CoA, necessary for lipid synthesis and nuclear histone acetylation (Morrish et al., 2010).

Of interest, an aberrant MYC expression in EBV-infect lymphoma cells has been seen, both in BL and other lymphomas (Bultema et al., 2009; Fish et al., 2014). The concomitance of EBV infection and MYC aberrant expression may suggest either a cooperative role, or a mutual compensation driving lymphomagenesis (e.g., early role for EBV and subsequent acquisition of MYC aberrations). In LCL EBV-infected cells, for example, EBNA2 activates *MYC* transcription (Kaiser et al., 1999) via the EBV super-enhancers (ESEs; Zhou et al., 2015; Liang et al., 2016), a cluster of genes binding to many different EBNAs and transcriptional factors playing a role in LCL proliferation and survival (Lo et al., 2017). EBNA3 and EBV miRNAs seem to play a role as well in this process, which is currently under investigation (Bajaj et al., 2008; Lo et al., 2017).

## Clinical Implications

Since metabolic shift is a consistent aberration differentiating neoplastic from normal cells, it is conceivable that interference with that process may represent an effective therapeutic strategy. EBV was demonstrated to be highly efficient in reprogramming infected cell metabolism toward amplified anabolism. For this reason, it represents a powerful strategy to identify key enzymes involved in this process and consider them as cancer metabolism drug targets (**Figure 2**; Galluzzi et al., 2013; Singh et al., 2015).

Because EBV was demonstrated to deregulate glycolysis, anti-glycolytic therapy might look as a promising opportunity in the management of EBV-related cancers (Xiao et al., 2014). Unfortunately, the inhibition of glycolytic enzymes may increase the risk of adverse events such as hypoglycorrhachia. To overcome this limit, it might be possible to target glucose transporters and glycolytic enzymes that are preferentially used by cancer cells compared with normal cells (Hay, 2016). Another possible solution is to use anti-diabetic drugs that do not cause hypoglycemia, such as metformin. Metformin has been found to induce G1 cell-cycle arrest and inhibit the proliferation of neoplastic cells (Anisimov et al., 2005; Libby et al., 2009; Cairns et al., 2011; Lo et al., 2013, 2015; Lei et al., 2017). The therapeutic value of metformin in solid tumors has been confirmed by many clinical trials and is worthy of further investigation in the context of hematological malignancies (Lo et al., 2013, 2015; Chae et al., 2016; Gong et al., 2016).

Regarding the induction of *HIF1A* expression, topotecan and bortezomib, two drugs that inhibit *HIF1A* translation and transactivation, respectively, have been FDA-approved for the treatment of lymphoid and solid cancers (Kummar et al., 2011; Lo et al., 2013, 2015, 2017; Raedler, 2015). Ganetespib, a drug that inhibits HIF1A stability, is currently being evaluated in a Phase III trial for solid tumors (Lo et al., 2013, 2015, 2017; Jhaveri and Modi, 2015) while novel HIF1A inhibitors are under development. Agents that interfere with some of the downstream effectors of HIF1A, including VEGF and carbonic anhydrase IX, have been designed as well. Carbonic anhydrase can, in fact, maintain the intracellular pH in a range that is compatible with cell proliferation while it lowers the microenvironmental, extracellular, pH conferring a survival advantage to tumor cells growing in a hypoxic and acidic microenvironment (Chiche et al., 2009). Agents decreasing the ROS levels in the cells, in particular superoxide dismutase mimetics, have also been shown to reduce HIF1A expression in experimental cancer settings (Batinic-Haberle et al., 2010).

The AMPK activator 5-aminoimidazole-4-carboxamide ribonucleotide formyl-transferase/IMP cyclohydrolase (ATIC, formerly named AICAR) has been shown to inhibit neoplastic cell growth and potentiate the cytotoxic effects of chemotherapeutic drugs in different kind of cancers (Rattan et al., 2005; Lo et al., 2013, 2015).

As for FAS, several FASN inhibitors have been investigated *in vitro* and xenograft studies and preliminary results show them to have both a killing tumor direct effect and a sensitizing effect to other cancer common therapies, e.g., 5-FU and trastuzumab (Pizer et al., 1998; Kridel et al., 2004; Vázquez et al., 2008).

## Conclusion

Oncoviruses, including EBV, can reprogram host cell metabolism to support viral persistence and tumor initiation. In the presence of pre-existing or acquired genetic mutations, the virus may indeed enable malignant transformation. In the case of some aggressive B-NHL (i.e., DLBCL and endemic BL), it is currently thought that genetic aberration such as MYC rearrangements would occur in an EBV-positive memory cell re-entering the germinal enter reaction. Remarkably, this model is compatible with “hit and run” hypothesis. In fact, despite allowing tumor initiation, viral particles can be lost during tumor progression, thus not diminishing the importance of viral contribution. Recent evidence shows that tumors apparently negative for EBV do carry reminiscence of a previous infections in terms of both residual viral DNA or miRNAs (Mundo et al., 2017). The metabolic shift as well as the anti-apoptotic phenotype would facilitate the complete acquisition of the malignant phenotype in EBV-infected cells.

Currently, a number of metabolism-interfering drugs are under evaluation in clinical trials, some of which have been approved by the FDA for cancer treatment. Hopefully, it will be possible to combine them with conventional chemotherapies, as well as with the newest targeted agents to increase anti-tumor efficacy while containing treatment toxicity. Lastly, the clear evidence that oncoviruses, and specially EBV, have a relevant role in the establishment of malignant lymphomas (Ambrosio et al., 2014a), should prompt current research to improve the vaccination strategies against these common pathogens as it happened for HPV.

## Author Contributions

PP, AW, MA, YA, and LL equally contributed to this article in terms of data collection, writing and critical revision. PP and LL coordinated the work.

## Conflict of Interest Statement

The authors declare that the research was conducted in the absence of any commercial or financial relationships that could be construed as a potential conflict of interest.
